# All-Polyethylene Tibial Component in Unicompartmental Knee Arthroplasty Offers Excellent Survivorship and Clinical Outcomes at Short-Term Follow-Up: A Multicenter Retrospective Clinical Study

**DOI:** 10.3390/medicina60091451

**Published:** 2024-09-04

**Authors:** Tommaso Bonanzinga, Federico Maria Adravanti, Umberto Vitale, Giuseppe Anzillotti, Francesco Iacono, Maurilio Marcacci

**Affiliations:** 1 IRCCS Humanitas Research Hospital, Via Manzoni 56, Rozzano, 20089 Milan, Italy; t.bonanzinga@gmail.com (T.B.); umberto.vitale@humanitas.it (U.V.); francesco.iacono@humanitas.it (F.I.); maurilio.marcacci@humanitas.it (M.M.); 2 Department of Biomedical Sciences, Humanitas University, Via Rita Levi Montalcini 4, Pieve Emanuele, 20072 Milan, Italy; 3 II Clinica Ortopedica e Traumatologica, IRCCS Istituto Ortopedico Rizzoli, 40136 Bologna, Italy; federicomaria.adravanti@ior.it

**Keywords:** unicompartmental knee arthroplasty, polyethylene tibial plateau, unilateral knee replacement, osteoarthritis

## Abstract

*Background and Objectives*: The ten-year survivorship of unicompartmental knee arthroplasty (UKA) is up to 96%, varying from implants and hospitals; however, most of registry studies do not distinguish between metal-back (MB) tibial implants and all-polyethylene (AP) tibial implants. The aim of the present retrospective clinical study was to analyze the clinical outcomes and survivorship of medial and lateral UKA with a newly designed all-polyethylene tibial plateau at short-term follow-up. *Materials and Methods*: A retrospective analysis of prospectively collected consecutive patients who underwent medial or lateral UKA with AP tibial plateau was conducted, with a minimum follow-up of 1 year. Primary outcomes were clinical score (VAS, OKS, and KOOS) variations from baseline up to the latest follow-up. Secondary outcomes were Likert scale variations from baseline to the follow-up, evaluation of the influence of demographic factors (age and BMI) at the time of surgery on the clinical outcomes, and evaluation of revision rate up to the last follow-up. *Results*: The final study population included 99 knees. The mean VAS score for the medial group significantly decreased from 7.61 ± 1.65 (pre-intervention) to 2.74 ± 2.26 (post-intervention). Similar improvements were registered for the OKS as well, for both the medial group (from 22.5 ± 12.6 to 36.6 ± 10.6, with a delta of 14.11 (10.05 to 18.17)) and the lateral group (from 22.6 ± 12.6 to 36.9 ± 11.8, with a delta of 14.24 (8.65 to 19.83)). Moreover, all the KOOS subscales reported an amelioration, both in medial UKA and lateral UKA. Furthermore, a logistic regression of delta VAS was performed in relation to the other clinical questionnaires and the demographic factors. For both medial and lateral UKAs, no statistically significant correlation was found between the VAS scale regression and the demographic factors. The survival rate free from any revision of the cohort at the latest follow-up was 96.32%. *Conclusions*: All-polyethylene tibial component in unicompartmental knee arthroplasty demonstrates significant improvements in clinical scores and a low failure rate at short-term follow-up.

## 1. Introduction

Osteoarthritis (OA) is considered a high-incidence disease responsible for the highest rate of disability among older adults and comprises up to 2.5 percent of the gross domestic products [1,2,3]. Although the last stages of the disease may involve the entire joint [4], it is not uncommon to observe the involvement of only one joint compartment. In fact, it is estimated that up to 50% of the population suffers from a unicompartmental disease, with the medial tibiofemoral compartment (27%) most affected, followed by the patellofemoral (18%) and, lastly, the lateral tibiofemoral (5%) [5]. It is still debated which is the most suitable option for patients with end-stage unicompartmental OA, but unicompartmental knee arthroplasty (UKA) has established itself over the years as a valid option in both young and active and older patients [6]. The ten-year survivorship of UKA is confirmed to be up to 96%, varying from implants and hospitals; however, most of the registry studies do not distinguish between metal-back (MB) tibial implants and all-polyethylene (AP) tibial implants [7,8]. While the AP was employed previously [9,10,11,12], in recent years, some surgeons have opted to discontinue its use in preference of the MB, considering it safer in terms of early risk of failure [13,14,15] and better in terms of clinical outcomes [16,17], especially in obese patients [18]. Sessa et al. reported that AP implants had greater survivorship but inferior outcomes compared to MB implants in 143 patients at long-term follow-up [19]. Other authors reported no differences between the AP and MB implants in terms of functional outcomes, quality of life, and survivorship [20,21], while Lustig et al. found excellent long-term results in a series of 172 AP tibial implant with minimal bone cuts [22], and Murray et al. found a survivorship of 68% at 25 years in a cohort of lateral UKA [23]. However, the overall evidence is limited by the paucity of well-conducted clinical trials describing clinical outcomes and survivorship of AP UKA implants [24]. Moreover, the advent of ultra-high molecular weight polyethylene (UHMWPE) in the last decade has pushed clinicians again towards the use of the AP tibial implant, with potential benefits given by the absence of a locking mechanism failure, the minimal backside wear, the reduction in implant cost, and a potentially more symmetrical load distribution compared to MB designs [25]. Indeed, a recent meta-analysis of randomized controlled trials (RCTs) comparing MB versus AP implants for unilateral knee OA showed how the clinical scores and survival of the implants was comparable between the two groups; however, the AP group outperformed the MB group in the radiostereometric analysis [26]. Hence, the aim of the present retrospective clinical study was to analyze the clinical outcomes and survivorship of medial and lateral UKA with a newly designed all-polyethylene tibial plateau (SLED^®^, Waldemar Link GmbH & Co. KG, Hamburg, Germany) at the short/mid-term follow-up.

## 2. Materials and Methods

This is a retrospective analysis of prospectively collected consecutive patients who underwent a medial or lateral UKA with AP tibial plateau (SLED^®^, Waldemar Link GmbH & Co. KG, Hamburg, Germany). The study was performed in two separate orthopedic centers both specialized in knee arthroplasty and sports medicine (Humanitas Research Hospital, Rozzano, Italy and Humanitas San Pio X, Milan, Italy). Written informed consent was obtained from all individual participants included in the study. The protocol was approved by the Ethical Committees/Internal Review Boards of each site and the study was conducted in agreement with either the Declaration of Helsinki (Tokyo, Venice, Hong Kong, and Somerset West amendments) (study n° 3156). 

From March 2017 to December 2021, UKA surgeries were performed by a single expert knee surgeon (MM). Data were collected from clinical records and stored in a limited access area with daily backup by one experienced knee surgeon (TB) and one researcher (FMA).

The inclusion criteria were as follows: aged 20–90 years, primary knee OA in medial or lateral compartment treated with a UKA with an AP tibial plateau, intact anterior cruciate ligament (ACL), and a minimum follow-up of 1 year after surgery.

The exclusion criteria were as follows: presence of contralateral compartment OA, presence of anterior OA, concomitant surgeries on the index knee, previous history of high tibial osteotomy on the affected knee, previous surgery for traumatic events in the index knee, presence of inflammatory arthropathies (e.g., rheumatoid arthritis), any known history of intra-articular or osseous infection of the index knee.

The surgical procedure is performed according to a minimally invasive technique, a quadriceps-sparing technique for the medial UKA, and a minimally invasive lateral approach for the lateral UKA, with a capsular approach with respect to the vastus lateralis. A tourniquet was used in all cases. The limb is placed in a thigh support with 45° flexion of the hip, and the leg is hanging down. It should be possible to flex the knee at least 120°. When using a medial incision, a lateral thigh support is needed. The surgery is performed with the surgeon in front of the flexed knee. The other leg is placed in a leg support, leaving plenty of space for the surgeon and the assistant. The skin and capsular incision were performed with knee flexed at 90° both in case of medial and lateral compartment.

In the postoperative settings, all patients were given conventional analgesic therapy such as Tramadol and Paracetamol. All the patients were given anti-thrombosis prophylaxis with Enoxaparine for at least 4 weeks, depending on comorbidity and anticoagulation status of the patient.

Patients began active knee movements (flexion and extension) and passive knee motion with motorized equipment (Kinetec) in the early morning after surgery. Full weight-bearing exercise carried out by ward physiotherapist and with the aid of two elbow crutches was started on the first postoperative day. Full extension and 90° flexion were the goal at discharge. The patients were monitored to ensure adequate wound healing within the first 2 weeks, and the stitches were removed 2 weeks after the surgery.

### 2.1. Outcomes

Primary outcome is as follows: clinical score variations from baseline up to latest follow-up (Pain Visual Analogue Scale (VAS) [27], Knee Injury and Osteoarthritis Outcome Score (KOOS) [28], and the Oxford Knee Score (OKS)) [29].

Secondary outcomes are as follows: Likert scale [30] administered at the latest follow-up; evaluation of the influence of demographic factors (age and body mass index (BMI)) at the time of surgery on the clinical outcomes; evaluation of revision rate up to the last follow-up.

Failure was defined as the need for any further surgical treatment on the index knee.

### 2.2. Data Analysis

Data were described as number and percentage, if categorical, or mean and standard deviation, if continuous and approximately Gaussian, or median and range, otherwise. Deltas between the pre- and post-procedure values were also described with their 95% confidence intervals (95%CI). Associations of covariates with delta VAS were explored with linear regression analysis. OKS and KOOS scales were also categorized for a simplification of interpretation. Differences among categories of age and BMI were explored with the Kruskal–Wallis test. A *p* value of less than 0.05 was considered statistically significant. All analyses were performed with STATA (15, StataCorpLLC, College Station, TX, USA).

## 3. Results

A total of 136 UKA surgeries were performed during the study period. Up to 37 patients could not complete the clinical questionnaires administered at follow-up due to personal reasons, so they were excluded from the analysis of the clinical outcomes, although they were included in the analysis of the revision rate. Hence, the final study population included 99 knees, with 90 affected by primary OA and 9 by secondary OA.

The mean age at surgery of the patients included was 62.6 ± 12.2 years. Of these patients, 51 (51.2%) were women and 48 were men (48.8%). The mean body mass index (BMI) was 25.7 ± 3.9 kg/m^2^. The minimum follow-up from surgery was 12 months, with a mean of 34.5 ± 14.0 months. The average latest follow-up was 65 months for medial UKA and 61 months for lateral UKA. No intra- or postoperative complications were reported in this series.

Among the finally included patients, 25 UKA (25.25%) were lateral, while 74 (74.75%) were medial. Demographic characteristics listed in Table 1.

The mean VAS score for the medial group significantly decreased from 7.61 ± 1.65 (pre-intervention) to 2.74 ± 2.26 (post-intervention), indicating a substantial reduction in pain. The delta (95% confidence interval [CI]) was calculated as −4.86 (−5.52 to −4.21). Similarly, the lateral group showed a significant decrease in the VAS score from 7.24 ± 1.98 to 2.52 ± 2.43, with a delta of −4.72 (−5.71 to −3.73). Similar improvements were registered for the OKS as well, for both the medial group (from 22.5 ± 12.6 to 36.6 ± 10.6, with a delta of 14.11 (10.05 to 18.17)) and the lateral group (from 22.6 ± 12.6 to 36.9 ± 11.8, with a delta of 14.24 (8.65 to 19.83)). Moreover, all the KOOS subscales reported an amelioration, both in the medial UKA and lateral UKA (Table 2).

However, when comparing the delta variation of the clinical scores examined, only KOOS pain and KOOS activity of daily living reported significant improvements in the medial cases compared to the lateral ones (*p* = 0.01 and *p* = 0.03, respectively).

Furthermore, a logistic regression of delta VAS was performed in relation to the other clinical questionnaires and the demographic factors in order to understand if a consistent and statistically significant pain was related to the other clinical scales or demographic factors such as age, BMI, and sex. For the medial UKA, statistically significant correlations were found with the Likert scale (*p* < 0.001), OKS (*p* = 0.013), KOOS symptoms (*p* = 0.001), KOOS pain (*p* < 0.001), and KOOS daily (*p* = 0.003), highlighting the importance of obtaining a significant pain reduction to also improve the other aspects of the patient’s life and global satisfaction after the surgery. For the lateral UKA, there was a statistically significant correlation with the Likert scale (*p* < 0.001). Moreover, for both medial and lateral UKA no statistically significant correlation was found between the VAS scale regression and the demographic factors (Table 3).

A further correlation analysis was conducted to evaluate the potential variations between the clinical scores, OKS and KOOS, in relation to age and BMI. There was no statistically significant correlation between the score values and age, both in the medial and lateral groups. Conversely, a higher BMI was associated to statistically significant better postoperative clinical scores for the OKS and KOOS symptoms and KOOS daily (*p* = 0.03, *p* = 0.02 and *p* = 0.01, respectively) (Table 4).

Moreover, the overall patient satisfaction grade was measured throughout the Likert scale, which was administered at the follow-up assessment. The average value of the scale was 2.1 ± 1.1 for the medial and 2.2 ± 1.3 for lateral, highlighting the high grade of patient satisfaction after the surgery.

The survival rate free from any revision of the cohort at the latest follow-up was 96.32%, with 5 revisions for tibial aseptic loosening out of 136 surgeries performed. In particular, all the revisions were related to the aseptic loosening of the implant with four cases of mobilization of the tibial plateau and one case related to the femoral component loosening.

## 4. Discussion

The main finding of the present study is that the all-polyethylene tibial component in unicompartmental knee arthroplasty demonstrated significant improvements in clinical scores after surgery at a mean follow-up of 34 months, with a 96.32% implant survival.

The previous literature demonstrated how the unexplained pain is a major UKA failure reason, accounting for up to 48% of all revisions [7]. In our study, we found an excellent improvement in pain, measured throughout the VAS scale, with 35 knees having no or forgettable pain (VAS scale 0 or 1) at the time of follow-up. A statistically significant correlation was also found between the improvement in the pain (VAS scale), the improvement in the symptoms, and the activity of daily living (both measured throughout the KOOS), especially for the medial group, meaning that the improvement in the pain influenced the activity and the personal feelings of the patient in relation to his pathology, bringing an advantage to several aspects of his life. Moreover, the improvement in the pain has been statistically linked to a mean high satisfaction of the patient, as our results have shown.

The results we retrieved are overall in line with the limited literature available for all-poly UKA [9,11,31,32]. Nonetheless, despite the positive results, various concerns and major debate have been raised in recent years regarding the use of AP tibial plateau UKAs, with many authors preferring metal-backed implants as they are considered safer with lower relative risk of early revision. Koh et al. and Scott et al. showed a high failure rate (11%) of the AP implants in the first 2 years after surgery mostly related to changes in bone mineral density below the tibial plateau resulting in residual pain [13,33]. Similarly, Batthacharya et al., with a mean follow-up of 44 months, found a revision rate of 8.8% of all-poly implants compared to the metal-backed option with a reduced satisfaction rate [13,33,34].

Conversely, other studies showed comparable revision rates for both implant designs with no significant differences, mostly for aseptic loosening, between the two groups [9,35,36]. When considering survivorship, we obtained results consistent to the current literature. Walker et al. obtained 100% at 2 years and Baur et al., with a mean follow-up of 3, 4 years, obtained 96.2% of survivorship. Moreover, for the OKS score, the former study obtained an average improvement (delta) from pre- to postoperative score of 13, while Baur et al. did not describe any average improvement but looked at the median postoperative OKS value which was 43 [37,38]. Furthermore, it is noteworthy how some evidence suggest that the use of all-polyethylene tibial components is able to offer, in case of failure, a chance of an easier revision due to the preservation of bone stock, compared to metal-backed implants, which may represent a significant advantage considering the crescent number of arthroplasties and, proportionally, revisions performed in this decade [13,39].

Accordingly, this study yielded similar results in terms of survivorship to Hawi et al., who affirmed that the survivorship of an all-polyethylene implant is more dependent on the surgical technique and on the single expert operator rather than implant design (all-poly or metal-backed) [20]. Additionally, Bush et al. highlighted the importance of the correct positioning of the UKA implant in order to achieve good clinical outcomes and survivorship. They compared expert surgeon’s UKAs with robotic-assisted ones, concluding that the former achieved or exceeded the robot in terms of implant alignment, strengthening the idea that an expert surgeon can influence implant outcomes, as per our study [40].

Lastly, patient selection is crucial, and contraindications to UKAs are often a source of debate. In fact, the literature agrees on the fundamental role of an accurate pre-operative assessment in order to exclude, with certainty, the involvement of the tibiofemoral compartment and the ACL deficiency, that are considered absolute contraindications [41] and would change the indication to one in favor of a total knee arthroplasty. There is, however, still modest disagreement on age limits for UKAs. Dalury et al. conducted a study on the clinical outcomes of UKAs in middle-aged (aged between 46- and 59-year-old) population yielding excellent results at follow-up, in disagreement with classical indications for UKAs, which required age >55 to obtain significant clinical results [42]. We found no statistically significant correlations between the clinical outcomes measured through OKS score and the age of the patients, and we believe that being aged < 60 should not be considered an absolute contraindication for UKA surgery [43].

We believe that our study embodies numerous strengths that contribute to its significance. This is one of the few studies available on the topic of assessing the patient’s satisfaction, which should be considered, above all, the clinical endpoint of any interventional procedure [44,45]. Moreover, we analyzed consecutive patients operated with the same implant by a single senior experienced orthopedic surgeon, which strongly limits the inter-operator variability.

Nonetheless, the present study is not free from limitations. Firstly, its retrospective nature did not allow us to assess the rate of amelioration of clinical scores at different time-points. Moreover, the short follow-up and the lack of radiological evaluation impeded the ability to assess the long-term survival of the prosthetic implant along with the OA progression [46], hence revision rate should be carefully considered. Lastly, the small sample size and lack of a control group consisting of patients who had undergone an MB implant with a polyethylene insert prevented any direct comparison between the two implants. Further long-term clinical studies with a prospective randomized design, a larger sample size, and a longer follow-up will be needed to assess the potential superiority of the AP implants over MB implants.

## 5. Conclusions

The all-polyethylene tibial component in unicompartmental knee arthroplasty demonstrates significant improvements in clinical scores with a 96.32% implant survival at a mean follow-up of 34 months.

## Figures and Tables

**Table 1:** Demographic characteristics of the included patient.

	Medial	Lateral	*p*
N	74	25	
Sex (men)	34 (45.95%)	14 (56.00)	0.384
Age at surgery	64.6 ± 12.2	56.7 ± 10.3	0.005
BMI *	26.1 ± 3.9	24.4 ± 3.6	0.052
MPTA (*n* = 72)	85.6 ± 2.8	88.3 ± 2.7	0.001
LDFA (*n* = 72)	89.4 ± 2.7	86.9 ± 3.2	0.002
Follow-up (months)	35.2 ± 14.5	32.6 ± 12.4	0.481

* BMI = body mass index; MPTA = medial proximal tibial angle; LDFA = lateral distal femoral angle.

**Table 2:** Pain Visual Analogue Score (VAS), Knee injury and Osteoarthritis Outcome Score (KOOS) subscales, Oxford Knee Score (OKS) variations from baseline to the follow-up assessment for medial and lateral unicompartmental knee arthroplasties.

	Medial			Lateral			
	Pre	Post	Delta (95%CI)	Pre	Post	Delta (95%CI)	*p* Delta M vs. Delta L
N	74	74	74	25	25	25	
VAS	7.61 ± 1.65	2.74 ± 2.26	−4.86 (−5.52; −4.21)	7.24 ± 1.98	2.52 ± 2.43	−4.72 (−5.71; −3.73)	0.818
Δ%			−61.4 ± 36.6			−66.7 ± 27.6	
OKS	22.5 ± 12.6	36.6 ± 10.6	14.11 (10.05; 18.17)	22.6 ± 12.6	36.9 ± 11.8	14.24 (8.65; 19.83)	0.729
Δ%			142.1 ± 208.5			123.9 ± 166.7	
TEGNER	2.28 ± 2.02	2.81 ± 1.25	0.53 (0.00; 1.06)	3.44 ± 2.87	3.56 ± 2.06	0.12 (−1.29; 1.53)	0.917
Δ%			77.2 ± 119.6			53.5 ± 116.4	
KOOS (symptoms)	48.6 ± 22.4	78.7 ± 16.8	30.09 (24.31; 35.88)	48.2 ± 23.2	74.7 ± 23.7	26.48 (18.76; 34.20)	0.509
Δ%			125.1 ± 199.3			81.3 ± 79.7	
KOOS (pain)	42.6 ± 20.6	80.8 ± 15.2	38.23 (32.92; 43.54)	52.1 ± 28.3	77.3 ± 24.5	25.24 (17.98; 32.50)	0.011
Δ%			184.2 ± 265.5			121.8 ± 180.5	
KOOS (daily)	44.4 ± 22.0	81.2 ± 16.6	36.81 (31.12; 42.50)	54.5 ± 29.7	79.6 ± 24.0	25.04 (17.33; 32.75)	0.031
Δ%			186.5 ± 348.1			71.6 ± 75.5	
KOOS (sport)	19.9 ± 20.0	54.7 ± 27.8	34.86 (27.75; 41.98)	31.4 ± 30.7	56.0 ± 32.6	24.60 (14.13; 35.07)	0.136
Δ%			318.2 ± 422.5			244.9 ± 356.1	
KOOS (QoL)	27.6 ± 18.6	60.3 ± 22.6	32.65 (26.76; 38.54)	34.6 ± 30.6	62.4 ± 28.4	27.72 (18.35; 37.09)	0.392
Δ%			220.4 ± 319.7			158.5 ± 198.7	

**Table 3:** Logistic regression of delta VAS (Visual Analogue Scale for Pain) in relation to demographic factors and clinical scores examined, divided for medial and lateral unicompartmental knee arthroplasties.

	Medial		Lateral	
	Coeff (95%CI)	*p*	Coeff (95%CI)	*p*
Sex (M)	0.13 (−1.19; 1.45)	0.844	−0.80 (−2.80; 1.21)	0.418
Age at surgery	0.00 (−0.05; 0.06)	0.948	−0.04 (−0.13; 0.06)	0.460
BMI	−0.03 (−0.20; 0.14)	0.758	−0.15 (−0.43; 0.12)	0.259
MPTA (*n* = 72)	0.11 (−0.19: 0.41)	0.463	0.08 (−0.41; 0.57)	0.734
LDFA (*n* = 72)	−0.13 (−0.44; 0.18)	0.404	−0.18 (−0.59; 0.22)	0.354
Follow-up (months)	−0.06 (−0.10; −0.01)	0.011	0.04 (−0.04; 0.12)	0.329
VAS preop	−1.02 (−1.34; −0.70)	<0.001	−0.47 (−0.95; 0.01)	0.052
OKS preop	0.06 (0.01; 0.11)	0.013	0.02 (−0.06; 0.10)	0.559
Tegner preop	0.18 (−0.14; 0.51)	0.260	−0.32 (−0.65; 0.01)	0.059
KOOS preop				
Symptoms	0.05 (0.02; 0.08)	0.001	−0.03 (−0.07; 0.02)	0.194
Pain	0.05 (0.03; 0.08)	<0.001	−0.02 (−0.05; 0.02)	0.328
Daily	0.04 (0.02; 0.07)	0.003	−0.01 (−0.04; 0.02)	0.538
Sport	0.02 (−0.01; 0.06)	0.164	−0.01 (−0.05; 0.02)	0.465
QoL	0.03 (−0.00; 0.07)	0.052	−0.02 (−0.05; 0.01)	0.181
Likert score	1.82 (1.40; 2.25)	<0.001	1.20 (0.63; 1.76)	<0.001

**Table 4:** Knee injury and Osteoarthritis Outcome Score (KOOS) and Oxford Knee Score (OKS) scores variations in relation to body mass index (BMI) and sex of the included patients.

	Medial			Lateral		
	All	Age	BMI	All	Age	BMI
*n*	74			25		
OKS postop						
<19	10 (13.51%)	70.7 ± 10.3	23.6 ± 1.7	5 (20.00%)	51.2 ± 10.2	24.9 ± 5.7
20–29	6 (8.11%)	58.0 ± 20.2	24.9 ± 3.8	2 (8.00%)	53.5 ± 2.1	22.2 ± 0.5
30–39	22 (29.73%)	65.8 ± 9.6	27.7 ± 4.3	4 (16.00%)	58.3 ± 19.3	24.3 ± 2.3
>40	36 (48.65%)	63.2 ± 12.2	26.0 ± 3.7	14 (56.00%)	58.6 ± 7.8	54.5 ± 3.5
*p*		0.179	0.030		0.560	0.851
KOOS symptoms						
<75	28 (37.84%)	63.5 ± 13.0	25.4 ± 3.8	11 (44.00%)	51.7 ± 8.4	24.0 ± 4.1
76–85	9 (12.16%)	58.9 ± 9.0	23.6 ± 1.9	3 (12.00%)	51.3 ± 11.7	22.8 ± 5.1
>85	37 (50.00%)	66.7 ± 12.1	27.2 ± 3.9	11 (44.00%)	63.1 ± 8.8	25.2 ± 2.9
*p*		0.193	0.022		0.016	0.543
KOOS pain						
<75	23 (31.08%)	65.6 ± 13.1	25.2 ± 2.9	10 (40.00%)	51.2 ± 8.7	24.3 ± 4.2
76–85	13 (17.57%)	67.1 ± 10.4	26.0 ± 4.5	1 (4.00%)	58	28.7
>85	38 (51.35%)	63.1 ± 12.3	26.7 ± 4.2	14 (56.00%)	60.5 ± 24.1	24.1 ± 3.3
*p*		0.534	0.321		0.089	0.489
KOOS daily						
<75	21 (28.38%)	65.0 ± 14.5	24.0 ± 2.7	9 (36.00%)	51.1 ± 9.2	24.6 ± 4.3
76–85	16 (21.62%)	67.1 ± 9.3	26.8 ± 3.9	2 (8.00%)	56.5 ± 6.4	23.3 ± 2.5
>85	37 (50.00%)	63.2 ± 12.1	27.0 ± 4.1	14 (56.00%)	60.3 ± 10.3	24.4 ± 3.5
*p*		0.567	0.010		0.113	0.909
KOOS sport						
<75	58 (78.38%)	64.1 ± 12.3	26.0 ± 4.1	16 (64.00%)	55.3 ± 12.2	24.4 ± 4.0
76–85	8 (10.81%)	65.3 ± 15.5	25.8 ± 3.2	3 (12.00%)	57.0 ± 1.0	24.1 ± 4.1
>85	8 (10.81%)	67.1 ± 8.7	27.1 ± 2.6	6 (24.00%)	60.2 ± 6.6	24.4 ± 3.0
*p*		0.802	0.758		0.636	0.993
KOOS QoL						
<75	57 (77.03%)	63.2 ± 12.2	26.0 ± 4.2	16 (64.00%)	54.4 ± 11.8	23.7 ± 3.9
76–85	4 (5.41%)	75.3 ± 14.8	27.6 ± 3.8	2 (8.00%)	63.5 ± 0.7	27.0
>85	13 (17.57%)	67.4 ± 10.1	26.1 ± 2.3	7 (28.00%)	59.9 ± 6.1	25.0 ± 3.2
*p*		0.105	0.719		0.332	0.430

## Data Availability

All the collected data are published in the present manuscript.
